# Supplementary material for: Hedgehog is relayed through dynamic heparan sulfate interactions to shape its gradient
Source: Nat Commun. 2023 Feb 10;14:758. doi: 10.1038/s41467-023-36450-y (PMC9918555; doi:10.1038/s41467-023-36450-y)
Supplement: Supplementary file 1 — Supplementary Information [file 41467_2023_36450_MOESM1_ESM.pdf]

**Supplemental information for**

# Hedgehog is relayed through dynamic heparan sulfate interactions to shape its gradient

**Fabian Gude, Jurij Froese, Dominique Manikowski, Daniele Di Iorio, Jean-Noël Grad, Seraphine Wegner, Daniel Hoffmann, Melissa Kennedy, Ralf P. Richter, Georg Steffes & Kay Grobe**

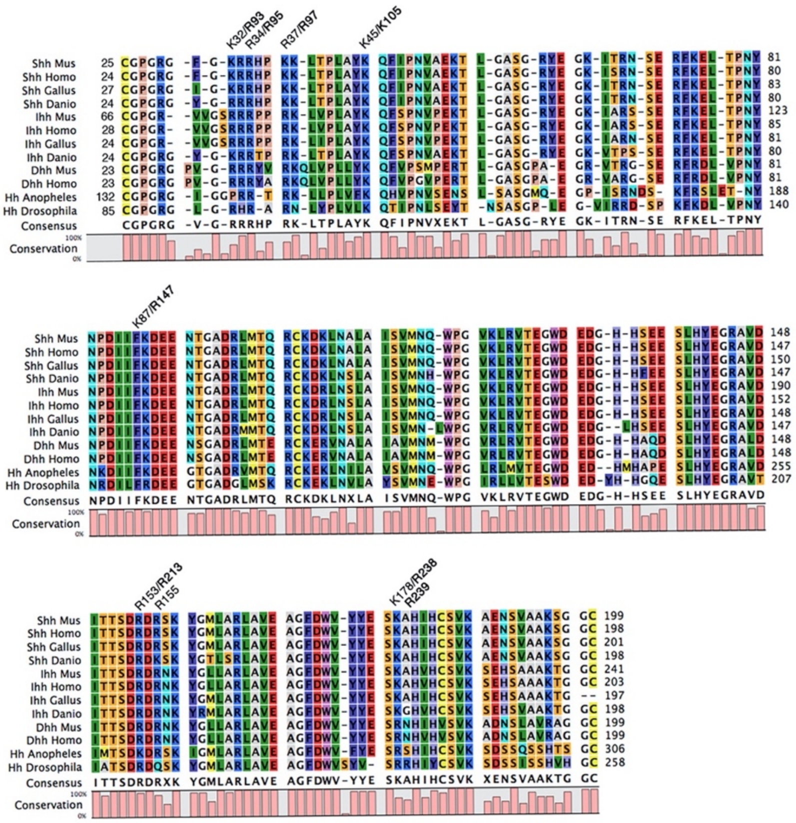


Supplementary figure 1**. HS-binding basic amino acids in vertebrate and invertebrate Hh family members are conserved.** Shh amino acids established to bind to HS and corresponding basic amino acids in *Drosophila* Hh (Fig. 1c,d) are labeled.

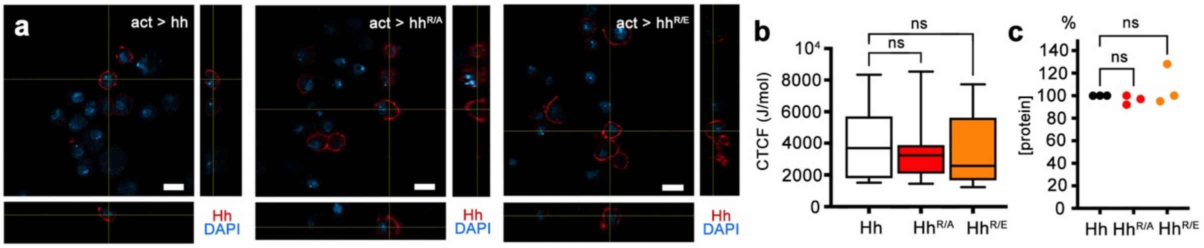


Supplementary figure 2. **Unimpaired protein autoprocessing and secretion. a** Confocal analysis of *Drosophila* S2 cells transfected with hh and hh$^{R/A}$ or hh$^{R/E}$ variants under actin-Gal4 control. All proteins were secreted and locate at the cell surface (red). Cells were stained with α-Hh antibodies (1:250) and Cy5-labeled α-rabbit IgG (1:300) under non-permeabilizing conditions. Representative results in orthogonal view are shown from experiments repeated at least three times. Scale bar: 10 µm. **b** Corrected total cell fluorescence (CTCF). Fluorescence at the cell surface was determined from fluorescence microscopy images using ImageJ. Box plots indicate median (middle line), 25$^{th}$, 75th percentile (box) and minimum/maximum values (whiskers). One-way ANOVA, Dunnett's multiple comparisons test, Hh: n=14, Hh$^{R/A}$: n=7, Hh$^{R/E}$: n=10. Medians and 25$^{th}$/75th percentiles: Hh: 3838 J/mol, 1795/5709 J/mol; Hh$^{R/A}$: 3728 J/mol, 2090/3896 J/mol; Hh$^{R/E}$: 3533 J/mol, 1664/5628 J/mol. F=0.05. p=0.94. **c** Hh variants were expressed in S2 cells and proteins secreted into the media detected by immunoblotting and quantified using ImageJ. One-way ANOVA, Sidak's multiple comparisons test, n=3 biologically independent samples were analyzed for each set. Mean values ± SD: Hh: 100 % ± 0.1 %, Hh$^{R/A}$: 96.4 % ± 4 %, Hh$^{R/E}$: 107.7 % ± 18 %. n.s: p>0.05, F=0.9, p=0.45. Source data are provided as a Source Data file.

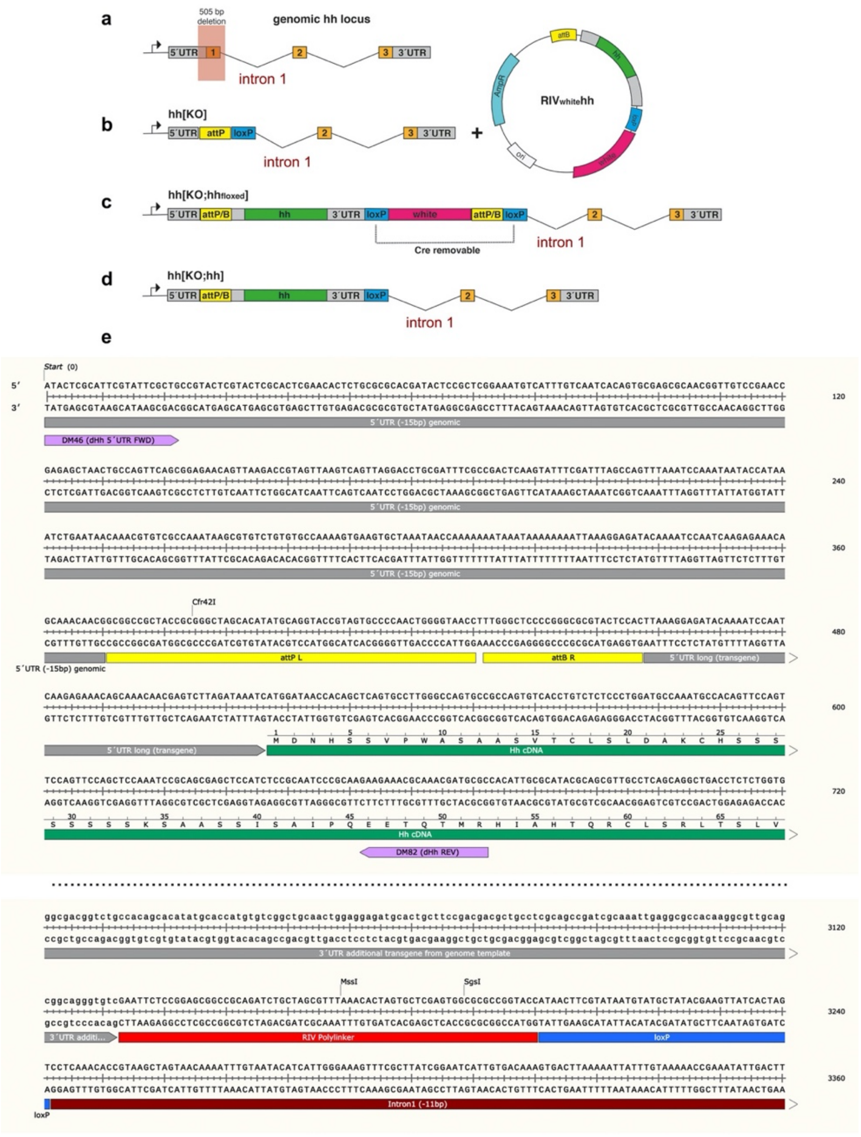


Supplementary figure 3. **Cloning strategy for the expression of transgenic hh and hh variant forms under endogenous promotor control. a** The targeted *hh* locus [1]. *Hh* coding exons 1-3 are labeled orange, 5′ and 3′ untranslated regions (UTRs) gray, and transgenic hh cDNA cloned into $RIV_{white}$hh green. The 505 bp *hh* coding region deleted by homologous recombination is indicated by the orange square. **b** The resulting hh[KO] allele after Cre-mediated excision of the loxP-flanked region of the targeting vector. **c** The hh[KO;hhfloxed] locus after reintegration of $RIV_{white}$hh. **d** The hh[KO;hh] locus after Cre-mediated RIV backbone excision represents one genotype studied in our work. **e)** Sequence confirmation of the hh[KO;hh] locus allowing for unimpaired transgenic *hh* expression under endogenous promotor control.

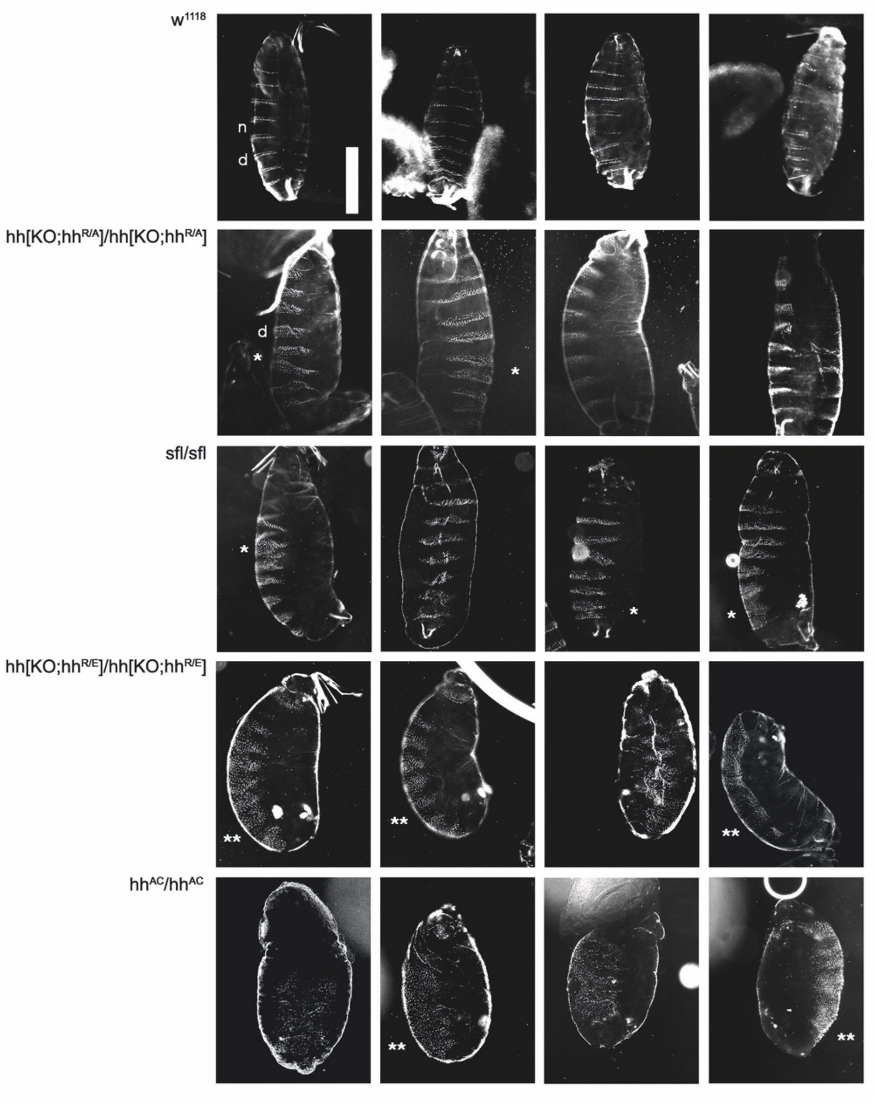


Supplementary figure 4. ***hh* null mutant embryonic phenotypes and phenotypes of null mutants for genes encoding HS or HS-modified core proteins are similar** [2, 3, 4, 5, 6, 7, 8, 9, 10, 11]. These similarities demonstrate that abolished direct interactions of $Hh^{R/A}$ and $Hh^{R/E}$ with HS cause these characteristic segment polarity phenotypes. Polarity phenotypes were consistently observed in three independent crosses and preparations. n: naked cuticle, d: denticle cuticle, * mirror image duplications of isolated parasegments, ** mirror image duplications of all parasegments (meaning that in each segment a defined fraction of the normal pattern is deleted and the remainder is duplicated and has reversed polarity). Scale bar: 100 μm.

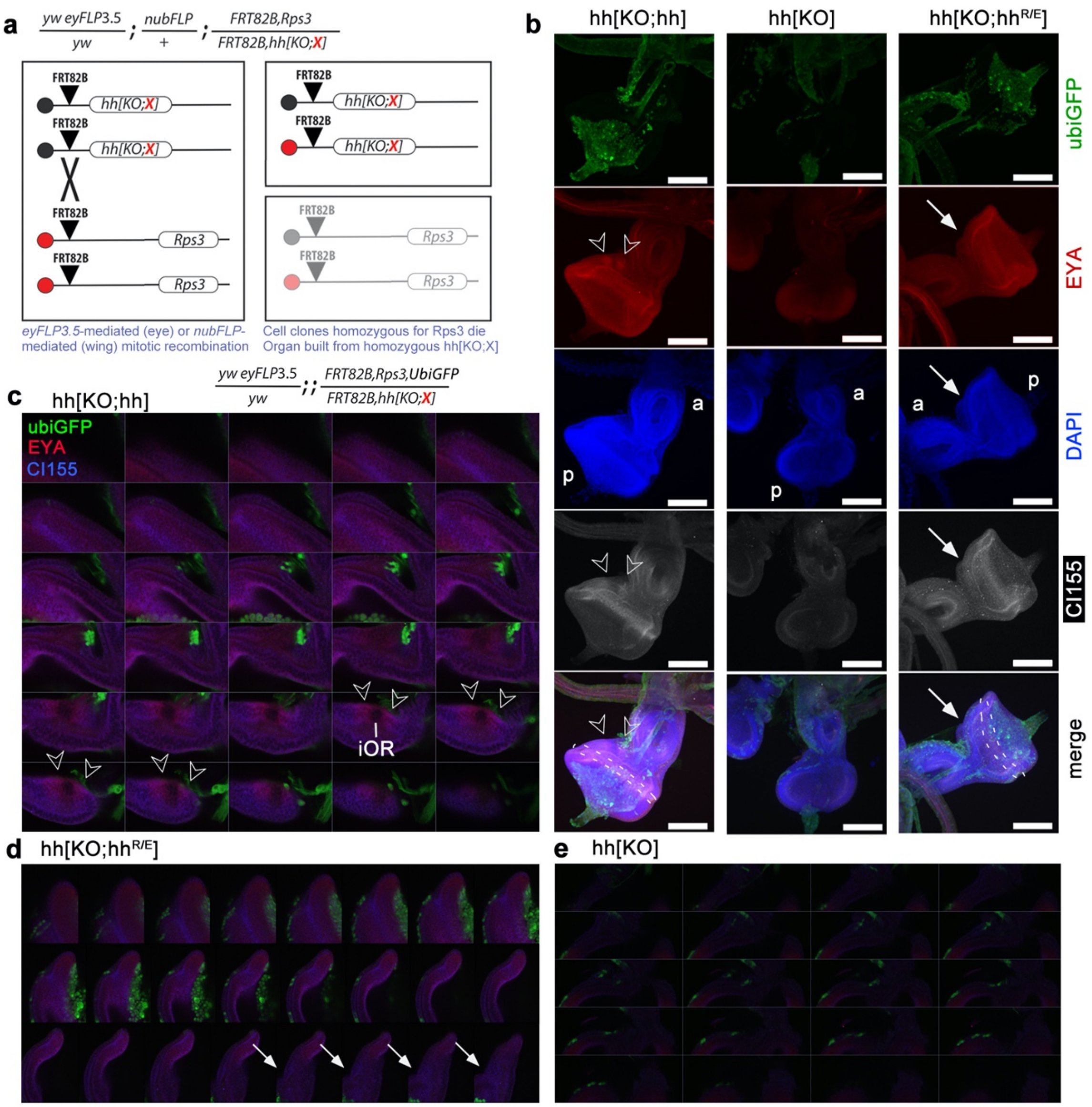


Supplementary figure 5. **Genetic basis of Minute mosaic analysis in wing and eye discs.** We conducted mosaic analyses to test the rescue capabilities of both hh variants while avoiding hh[KO;hh$^{R/A}$];hh[KO;hh$^{R/A}$] or hh[KO;hh$^{R/E}$];hh[KO;hh$^{R/E}$] lethality (Fig. 2). **a** We used clonal analysis with the "Minute" technique that is established for all major chromosome arms in *Drosophila*. Here, the counter chromosome of the modified hh[KO;X] allele bears a mutation in Rps3 (Ribosomal protein S3, a component of the small subunit of cytoplasmic ribosomes) on *Drosophila* chromosome 3R. Because Minute mutations are dominant, cells heterozygous for the Rps3 mutation have a competitive disadvantage, as ribosomal capacity is rate limiting for protein biosynthesis of the cell, and cell clones homozygous for Rps3 die after induction of mitotic recombination and are eliminated from

the tissue. As a consequence, the organ in the clonal area is built from the homozygous hh[KO;X] cell population, which is not proliferatively hampered by Rps3. This, in turn, generates large patches of tissue with homogeneous expression of the mutant allele. In our experiments, the Flippase source used was eyFLP3.5 or nubFLP or a combination of both. If needed, depletion of cell clones homozygous for Rps3 can be visualized during development with the addition of an UbiGFP on the same arm as Rps3 (**b** and **c** and the **Supplemental movie 1)**. We found the Minute approach for our question superior to other clonal analysis techniques like MARCM that can also address cell autonomy of gene function, yet clone sizes are smaller and there is no twin spot elimination. The analysis of *hh* signaling range, however, is by definition not a question of cell autonomy, hence as large and uniform clones as possible are the favorable option in this case and, given the survival of animals with large Minute clones to eclosion, permits the end point analysis in the imago. Moreover, *en route* phenomena like small and varying clone sizes, effects of cell competition, possible mixture of hh signal from non-uniform sources like side-by-side stripes of homozygous and heterozygous cells can be completely factored out, and the net overall effect of hh signaling range can therefore be scored. The X in hh[KO;X] stands for no inserted hh (clones are homozygous for hh[KO], representing *hh* null cells), for hh[KO;hh] or for hh variants hh[KO;$hh^{R/A}$] or hh[KO;$hh^{R/E}$], all expressed under endogenous promotor control in the eye disc (eyFLP3.5) or in the wing disc (nubFLP). Top: Genotype in which clonal eye disc or wing disc analysis was conducted. Because flies express eyFLP3.5 from chromosome 1 and nubFLP from chromosome 2, both analyses can be performed in the same fly. **b** In wandering L3 larvae, all tissue with eyFLP3.5 expression has proliferated and is built from homozygous hh variant clonal tissue alone after the initial mitotic recombination event, as shown by the lack of UbiGFP expression in the disc proper (the entire part that gives rise to the eye - including the area of the prospective ocelli (white arrowheads, also see Supplementary movie 1)). GFP signals in the eye disc flooring stem from CNS derived GFP-positive heterozygous glia that have undergone migration from the brain through the optic stalk to get in contact with the nascent photoreceptor cells (also see [12]). Other GFP positive cells are superficial on top of the imaginal disc (also see Supplementary movie). Other contributors to GFP positive tissue surrounding the disc proper surface are the Bolwig nerve and other tissue, most likely peripodial and developing muscle cells. Expression of the retinal determination gene *eyes absent* (*eya*) and elevated levels of Cubitus interruptus (Ci155) label developing ocelli (white arrowheads) in hh[KO;hh] discs, but are absent in discs of hh variants hh[KO] or hh[KO;$hh^{R/E}$]. Instead, we observe indentations (white arrow) in their corresponding disc regions. The morphogenetic furrow is marked by the dashed line. a: anterior, p: posterior, scale bar: 100 μm. Morphological and gene expression changes were consistently observed in seven independent preparations of the respective genotypes. **c** Serial magnifications of the ocellar region of hh[KO;hh] shown

in (b). Arrowheads indicate prospective ocelli, iOR: interocellar region (Fig. 3b). **d,e** Serial magnifications of the ocellar regions of hh[KO;hh$^{R/E}$] and hh[KO] shown in (b), respectively. White arrows in (d) refer to the indent also marked in (b) hh[KO;hh$^{R/E}$].

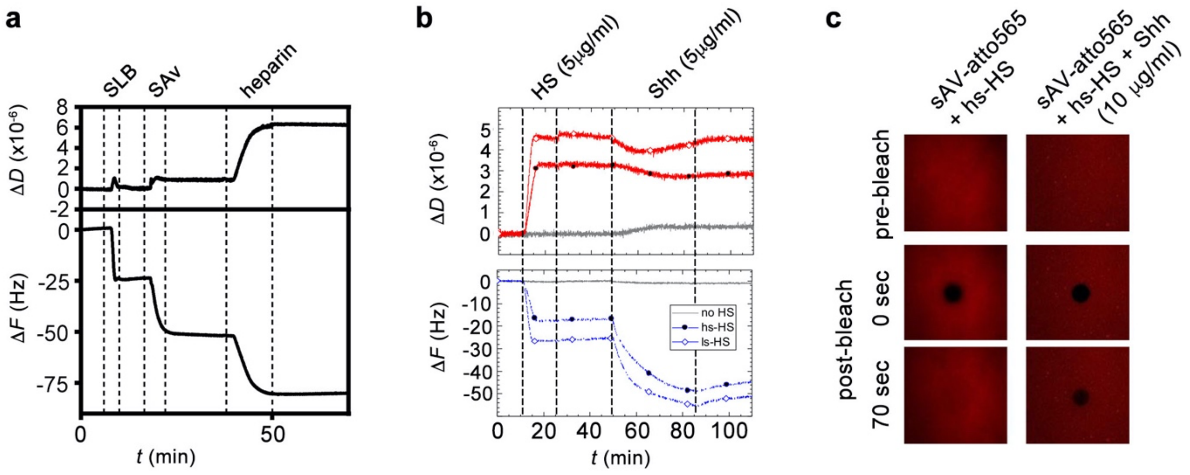


Supplementary figure 6. **Preparation and validation of a heparin/HS cell surface model matrix for QCM-D and FRAP analyses. a** Representative QCM-D data displaying the observed frequency ($\Delta F$) and dissipation ($\Delta D$) shifts during assembly of the SLB, the SAv monolayer and the film of end-attached heparin on the sensor's silica surface. Start and duration of sample incubations are indicated by dashed vertical lines and a label on top of the graph. At all other times, the surface was exposed to wash buffer A. The formation of the heparin/HS model matrix on the QCM sensor was always followed in real-time prior to the protein incubation assays shown in Fig. 5 and Fig. 6, to validate proper surface functionalization. **b** Representative QCM-D data (presented analogous to A) for the formation of model matrices with high-sulfated HS (hs-HS; 1.6 sulfates/disaccharide; blue and red lines with circles) and low-sulfated HS (ls-HS; 1.0 sulfate/disaccharide; blue and red lines with diamonds), respectively, and subsequently Shh interaction. Start and duration of sample incubations are indicated by dashed vertical lines and a label on top of the graph. At all other times, the surface was exposed to Hepes buffer (10 mM HEPES pH 7.4, 150 mM NaCl). Note the overall similarity in Shh binding to both HS forms and to heparin (Fig. 5): Shh binding is pronounced, mostly stable upon a wash in buffer, and accompanied by a decrease $\Delta D$. However, the decrease in dissipation shift is less pronounced for HS ($\Delta D \approx 0.6 \times 10^{-6}$) than for heparin ($\Delta D \approx 2.0 \times 10^{-6}$). A control without HS (grey lines without symbols) shows negligible responses confirming Shh binding is specific for HS. **c** Representative fluorescence micrographs displaying FRAP results (acquired as described in [13]) on hs-HS coated surfaces. Shown are bleach areas before the bleaching process (pre-bleach) and at 0 s

and 70 s after the bleaching (post bleach), as indicated. Image size: 100 µm. The fluorescence is almost fully recovered for a bare HS layer after 70 s, indicating rapid diffusion of the SAv-anchored HS (SAv-atto565 was here used as mobility tracer), Shh/HS layers retain a substantial amount of bleaching, consistent with reduced diffusivity of HS due to Shh-mediated cross-linking. ls-HS was provided by Celsus Laboratories (Cincinatti, OH, USA), and hs-HS was purified and provided by Hughes Lortat-Jacob [13] (Institut de Biologie Structurale, Université Grenoble Alpes, Genoble, France). ls-HS and hs-HS were derived from porcine intestinal mucosa. The average level of sulfation was determined by disaccharide analysis (by Romain Vivès, Institut de Biologie Structurale, Université Grenoble Alpes, Genoble, France). The average size of the surface-anchored HS and heparin chains was estimated at 8 to 10 kDa from the analysis of $\Delta D/-\Delta f$ data as described in [14].

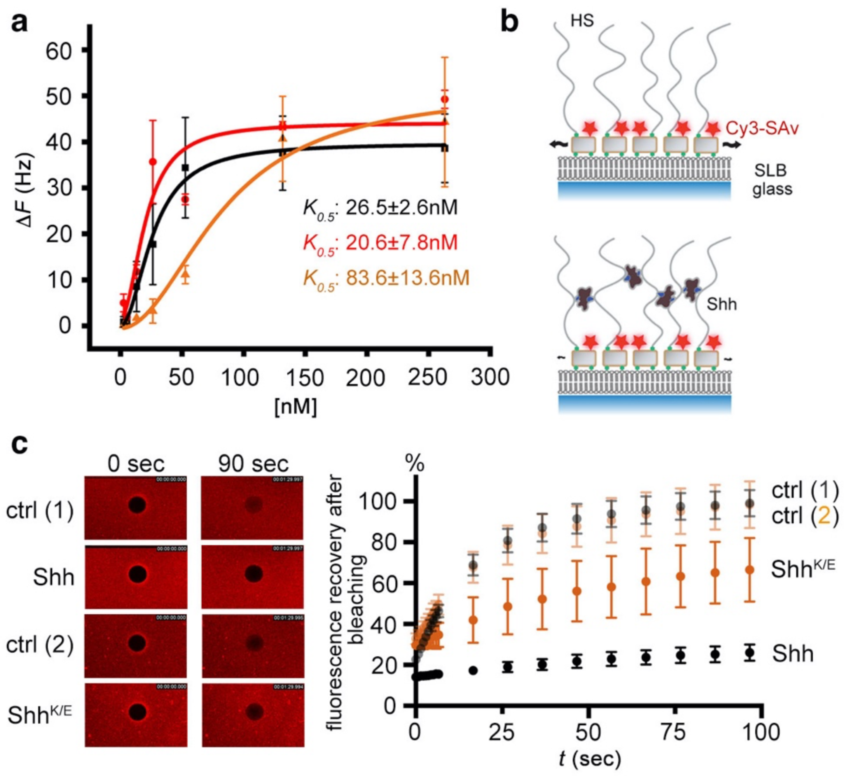


Supplementary figure 7**. ShhK/E binds HS, but HS cross-linking by the protein is impaired**. **a** Hill fit of QCM-D binding data obtained at different protein concentrations. While the concentration of half-maximal binding $K_{0.5}$ of $Shh^{K/E}$ exceeded that of Shh by a factor of about 2, the $K_D$ of $Shh^{K/A}$ was not changed in this assay. This might be explained by just one basic Shh amino acid being changed into a neutral alanine whereas two arginines are exchanged in *Drosophila* Hh. Source data are provided as a Source Data file. **b**

Preparation of heparin-functionalized surfaces for FRAP analyses of SLB-inserted heparin chain mobility in the presence or absence of protein. As one modification to the protocol used for QCM-D, streptavidin was fluorescently labeled (Cy3-SAv), allowing us to follow lateral HS movement. **c** Left: Representative fluorescence micrographs displaying the FRAP results. Shown are bleach areas at 0 s and post-bleach areas at 90 s. Ctrl: no added protein. Right: Quantitative analysis of FRAP measurements in terms of mobile fraction (%). The mean ± SD of three measurements is shown. Source data are provided as a Source Data file.

## Supplementary Tables

Supplementary Table 1. **Distribution of the distances (nm) between the center of mass of the two HS-binding sites, HS1 and HS2.**

| | Min | 1st Qu | Median | Mean | 3rd Qu | Max |
|---|---|---|---|---|---|---|
| 3m1n | 2.319 | 2.628 | 2.713 | 2.743 | 2.858 | 3.149 |
| Model M | 2.330 | 2.660 | 2.767 | 2.743 | 2.840 | 3.097 |
| Model S | 2.366 | 2.665 | 2.735 | 2.743 | 2.816 | 3.133 |

Min: minimum, Max: maximum, Qu: quartile

Supplementary Table 2. **P-values of distance comparisons (Wilcoxon test) between center of mass of HS1 and HS2 sites.** ShhN and Hh structures are included.

| | Model M (HhN) | Model S (HhN) |
|---|---|---|
| 3m1n | 0.13 | 0.39 |
| Model M (HhN) | - | 0.09 |

Supplementary Table 3. **Electrostatic potentials computed by Epitopsy commit revision d283db1.** $\Delta\Delta G^{elec}$ values (in cal per mol) were calculated as the difference of $\Delta G^{elec}$ between each respective mutant protein and its corresponding wild-type protein. The data correspond to the plots shown in Figure 1e.

| Model | Mutant | $\Delta\Delta G^{elec}$ |
|---|---|---|
| Model S | $Hh^{R/A}$ | 21.2 |
| Model S | $Hh^{R/E}$ | 29.7 |
| Model S | $Hh^{CW/A}$ | 43.0 |
| Model S | $Hh^{R/A;CW/A}$ | 65.0 |
| Model M | $Hh^{R/A}$ | 24.3 |
| Model M | $Hh^{R/E}$ | 33.4 |
| Model M | $Hh^{CW/A}$ | 31.4 |
| Model M | $Hh^{R/A;CW/A}$ | 54.4 |
| PDB 3m1n | $Shh^{K/A}$ | 22.5 |

| | | |
|---|---|---|
| PDB 3m1n | $Shh^{K/E}$ | 34.8 |

**Supplementary methods 1**

We have analyzed protein content of Hh and $Hh^{R/A}$ at times 0h, 3h, 6h, and 12h. Each measurement was done in triplicate. Protein content is given as fraction of initial content.

There is a clear decay of protein over 12h. Points for Hh and $Hh^{R/A}$ are not clearly separated though Hh may decay somewhat faster than $Hh^{R/A}$ (Fig 1). If we had a simple exponential decay, the points should lie on a straight line in a log-linear plot. This is not the case (Fig 2). In particular, there is a super-exponential drop around 3h.

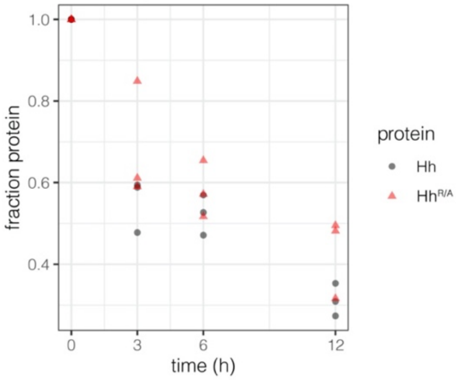


Figure 1: Fraction of remaining Hh and $Hh^{R/A}$ content, relative to the starting content. Both axes are scaled linearly.

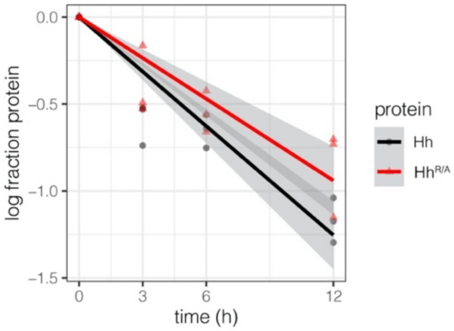


Figure 2: Fraction of remaining Hh and $Hh^{R/A}$ content, relative to the starting content. The vertical axis is scaled logarithmically. The two lines represent linear models fitted to the Hh (red) and $Hh^{R/A}$ (black) data. Gray areas are 95% confidence

intervals. The origins (corresponding to 100% protein) were assumed to be fixed in the fit.

Model: We assumed that for each time point (3h, 6h, 12h) and protein (Hh and $Hh^{R/A}$), the fractions $y_i$ of remaining protein $i = 0$ (for Hh) or 1 (for $Hh^{R/A}$) follow a beta distribution with average $\mu$ and width $\phi$,

$$f(y_i) = \frac{{y_i}^{\mu\phi-1}(1-y_i)^{(1-\mu)\phi-1}}{B(\mu\phi,(1-\mu)\phi)} \qquad (1)$$

where $B$ is the Beta function. We further assume that the mean fraction $\mu$ can be modelled as a generalized linear model:

$$\mu = \text{logistic}(\alpha + \beta x_j), \qquad (2)$$

with intercept $\alpha$, slope $\beta$ and an indicator variable $x_j$ that is 0 for Hh measurements and 1 for $Hh^{R/A}$ measurements. If there was a significant difference between Hh and $Hh^{R/A}$ decay dynamics, the slope $\beta$ would be clearly different from zero.

To quantify the probability distribution of $\beta$, we use a Bayesian model with Eqs 1 and 2 as likelihood, and the following priors:

$$\alpha \sim \text{Normal}\,(0, 2.5) \qquad (3)$$
$$\beta \sim \text{Normal}\,(0, 2.5) \qquad (4)$$
$$\phi \sim \text{Exponential}\,(1). \qquad (5)$$

The model was fitted with R package rstanarm [15]. Visual posterior predictive checks were performed to confirm agreement of model predictions with observed data. Result: The marginal posteriors of the slope parameter $\beta$ are all overlapping with zero (Fig.3), i.e. we cannot see significant differences in the decay of Hh and $Hh^{R/A}$.

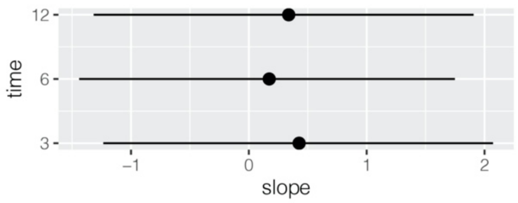


Figure 3: Marginal posterior probability of slope parameter $\beta$ for 3h, 6h, and 12h. The dots and bars represent the median and 95% intervals, ranging from the 2.5%to

the 97.5% quantile. Quantitatively, the fractions of the marginal posterior greater than zero are 0.72, 0.59, and 0.68 for 3h, 6h, and 12h, respectively.

## References:


1. Baena-Lopez LA, Alexandre C, Mitchell A, Pasakarnis L, Vincent JP. Accelerated homologous recombination and subsequent genome modification in Drosophila. *Development* **140**, 4818-4825 (2013).

2. Han C, Belenkaya TY, Wang B, Lin X. Drosophila glypicans control the cell-to-cell movement of Hedgehog by a dynamin-independent process. *Development* **131**, 601-611 (2004).

3. Williams EH, Pappano WN, Saunders AM, Kim MS, Leahy DJ, Beachy PA. Dally-like core protein and its mammalian homologues mediate stimulatory and inhibitory effects on Hedgehog signal response. *Proc Natl Acad Sci U S A* **107**, 5869-5874 (2010).

4. Takeo S, Akiyama T, Firkus C, Aigaki T, Nakato H. Expression of a secreted form of Dally, a Drosophila glypican, induces overgrowth phenotype by affecting action range of Hedgehog. *Dev Biol* **284**, 204-218 (2005).

5. Ayers KL, Gallet A, Staccini-Lavenant L, Therond PP. The long-range activity of Hedgehog is regulated in the apical extracellular space by the glypican Dally and the hydrolase Notum. *Dev Cell* **18**, 605-620 (2010).

6. Gallet A, Staccini-Lavenant L, Therond PP. Cellular trafficking of the glypican Dally-like is required for full-strength Hedgehog signaling and wingless transcytosis. *Dev Cell* **14**, 712-725 (2008).

7. Bellaiche Y, The I, Perrimon N. *Tout-velu* is a *Drosophila* homologue of the putative tumour suppressor EXT-1 and is needed for Hh diffusion. *Nature* **394**, 85-88 (1998).

8. The I, Bellaiche Y, Perrimon N. Hedgehog movement is regulated through *tout velu*-dependent synthesis of a heparan sulfate proteoglycan. *MolCell* **4**, 633-639 (1999).

9. Bornemann DJ, Duncan JE, Staatz W, Selleck S, Warrior R. Abrogation of heparan sulfate synthesis in Drosophila disrupts the Wingless, Hedgehog and Decapentaplegic signaling pathways. *Development* **131**, 1927-1938 (2004).

10. Han C, Belenkaya TY, Khodoun M, Tauchi M, Lin X. Distinct and collaborative roles of Drosophila EXT family proteins in morphogen signalling and gradient formation. *Development* **131**, 1563-1575 (2004).

11. Takei Y, Ozawa Y, Sato M, Watanabe A, Tabata T. Three Drosophila EXT genes shape morphogen gradients through synthesis of heparan sulfate proteoglycans. *Development* **131**, 73-82 (2004).

12. Franzdottir SR, Engelen D, Yuva-Aydemir Y, Schmidt I, Aho A, Klambt C. Switch in FGF signalling initiates glial differentiation in the Drosophila eye. *Nature* **460**, 758-761 (2009).

13. Migliorini E*, et al.* Cytokines and growth factors cross-link heparan sulfate. *Open Biol* **5**, (2015).

14. Srimasorn S*, et al.* A quartz crystal microbalance method to quantify the size of hyaluronan and other glycosaminoglycans on surfaces. *Sci Rep* **12**, 10980 (2022).

15. Goodrich B, Gabry J, Ali I, Brilleman S. rstanarm: Bayesian applied regression modeling via Stan. R package version 2.17.4 *https://mc-stanorg/*, (2018).
