# Supplementary material for: Hedgehog is relayed through dynamic heparan sulfate interactions to shape its gradient
Source: Nat Commun. 2023 Feb 10;14:758. doi: 10.1038/s41467-023-36450-y (PMC9918555; doi:10.1038/s41467-023-36450-y)
Supplement: Supplementary file 3 — Description of Additional Supplementary Information [file 41467_2023_36450_MOESM3_ESM.pdf]

Description of Additional Supplementary Material

Supplementary movie 1

Lack of UbiGFP expression in the disc proper confirms that the largest extend of clonal tissue is derived from the hh allele bearing twin spots, while the Minute twin spot has been eliminated. GFP signals in the eye disc flooring stem from CNS-derived GFP-positive heterozygous glia that have undergone migration to get in contact with the nascent photoreceptor cells. Shown in red is the retinal determination gene eyes absent (eya) and in white elevated levels of Cubitus interruptus (Ci155).
